# The impact of age on genetic testing decisions in amyotrophic lateral sclerosis

**DOI:** 10.1093/brain/awac279

**Published:** 2022-09-27

**Authors:** Puja R Mehta, Alfredo Iacoangeli, Sarah Opie-Martin, Joke J F A van Vugt, Ahmad Al Khleifat, Andrea Bredin, Lynn Ossher, Peter M Andersen, Orla Hardiman, Arpan R Mehta, Pietro Fratta, Kevin Talbot, Project MinE ALS Sequencing Consortium, Nazli A Başak, Philippe Corcia, Philippe Couratier, Mamede de Carvalho, Vivian Drory, Jonathan D Glass, Marc Gotkine, John E Landers, Russell McLaughlin, Jesus S Mora Pardina, Karen E Morrison, Monica Povedano, Christopher E Shaw, Pamela J Shaw, Vincenzo Silani, Nicola Ticozzi, Philip Van Damme, Leonard H van den Berg, Jan H Veldink, Patrick Vourc’h, Markus Weber, Ammar Al-Chalabi

**Affiliations:** UCL Queen Square Motor Neuron Disease Centre, Department of Neuromuscular diseases, UCL Queen Square Institute of Neurology, London, WC1N 3BG, UK; Maurice Wohl Clinical Neuroscience Institute, Institute of Psychiatry, Psychology and Neuroscience, King’s College London, London, SE5 9RX, UK; Maurice Wohl Clinical Neuroscience Institute, Institute of Psychiatry, Psychology and Neuroscience, King’s College London, London, SE5 9RX, UK; Department of Biostatistics and Health Informatics, Institute of Psychiatry, Psychology and Neuroscience, King’s College London, London, SE5 8AF, UK; National Institute for Health Research Biomedical Research Centre and Dementia Unit at South London and Maudsley NHS Foundation Trust and King’s College London, London, SE5 8AF, UK; Maurice Wohl Clinical Neuroscience Institute, Institute of Psychiatry, Psychology and Neuroscience, King’s College London, London, SE5 9RX, UK; Department of Neurology, UMC Utrecht Brain Center, University Medical Center Utrecht, Utrecht University, Utrecht, 3584 CG, The Netherlands; Maurice Wohl Clinical Neuroscience Institute, Institute of Psychiatry, Psychology and Neuroscience, King’s College London, London, SE5 9RX, UK; Maurice Wohl Clinical Neuroscience Institute, Institute of Psychiatry, Psychology and Neuroscience, King’s College London, London, SE5 9RX, UK; Nuffield Department of Clinical Neurosciences, University of Oxford, Oxford, OX3 9DU, UK; Department of Clinical Science, Neurosciences, Umeå University, Umeå, SE-901 87, Sweden; Academic Unit of Neurology, Trinity College Dublin, Trinity Biomedical Sciences Institute, Dublin, D02 R590, Republic of Ireland; Department of Neurology, Oxford University Hospitals NHS Foundation Trust, Oxford, OX3 9DU, UK; Euan MacDonald Centre for MND Research, University of Edinburgh, Edinburgh, EH16 4SB, UK; UCL Queen Square Motor Neuron Disease Centre, Department of Neuromuscular diseases, UCL Queen Square Institute of Neurology, London, WC1N 3BG, UK; Nuffield Department of Clinical Neurosciences, University of Oxford, Oxford, OX3 9DU, UK; Maurice Wohl Clinical Neuroscience Institute, Institute of Psychiatry, Psychology and Neuroscience, King’s College London, London, SE5 9RX, UK; National Institute for Health Research Biomedical Research Centre and Dementia Unit at South London and Maudsley NHS Foundation Trust and King’s College London, London, SE5 8AF, UK; Department of Neurology, King’s College Hospital, London, SE5 9RS, UK

**Keywords:** amyotrophic lateral sclerosis, motor neuron disease, genetic testing, age of onset, genetic counselling

## Abstract

Amyotrophic lateral sclerosis (ALS) is a heterogeneous neurodegenerative syndrome. In up to 20% of cases, a family history is observed. Although Mendelian disease gene variants are found in apparently sporadic ALS, genetic testing is usually restricted to those with a family history or younger patients with sporadic disease. With the advent of therapies targeting genetic ALS, it is important that everyone treatable is identified. We therefore sought to determine the probability of a clinically actionable ALS genetic test result by age of onset, globally, but using the UK as an exemplar.

Blood-derived DNA was sequenced for ALS genes, and the probability of a clinically actionable genetic test result estimated. For a UK subset, age- and sex-specific population incidence rates were used to determine the number of such results missed by restricting testing by age of onset according to UK’s National Genomic Test Directory criteria.

There were 6274 people with sporadic ALS, 1551 from the UK. The proportion with a clinically actionable genetic test result ranged between 0.21 [95% confidence interval (CI) 0.18–0.25] in the youngest age group to 0.15 (95% CI 0.13–0.17) in the oldest age group for a full gene panel. For the UK, the equivalent proportions were 0.23 (95% CI 0.13–0.33) in the youngest age group to 0.17 (95% CI 0.13–0.21) in the oldest age group. By limiting testing in those without a family history to people with onset below 40 years, 115 of 117 (98% of all, 95% CI 96%–101%) clinically actionable test results were missed.

There is a significant probability of a clinically actionable genetic test result in people with apparently sporadic ALS at all ages. Although some countries limit testing by age, doing so results in a significant number of missed pathogenic test results. Age of onset and family history should not be a barrier to genetic testing in ALS.

## Teaser

Some countries limit genetic testing in ALS to individuals with a family history or younger age of onset. However, Mehta, Iacoangeli *et al.* show that doing so results in a significant number of clinically actionable genetic test results being missed. Age of onset and family history should not be a barrier to genetic testing in ALS.

## Introduction

Amyotrophic lateral sclerosis (ALS) is a neurodegenerative disease, primarily affecting upper and lower motor neurons,^1^ with a lifetime risk of 1 in 300 people.^2^ The median survival is 30 months from symptom onset, with death typically resulting from neuromuscular respiratory failure. Onset is usually later in life, with the mean age of onset in population studies being 65, consistent with a multistep disease process involving a combination of sequential genetic and environmental factors.^3^ There is mounting evidence that for those with an identified genetic basis to their ALS, effective treatment may soon be possible.^4,5^

In up to 20% of people, a family history of ALS is obtained, usually inherited as a dominant trait. Genetic and pathological overlap with some forms of frontotemporal dementia is seen.^6^ The genetic cause of about 80% of familial ALS has now been identified,^1^ with the four commonest involved genes in the UK being *C9orf72*, *SOD1*, *TARDBP* and *FUS*. All of the genes implicated in familial ALS have also been reported mutated in sporadic ALS,^7^ and it is now widely accepted that apparently sporadic ALS may have a genetic basis.^8^ To that end, a better distinction may be between primarily genetic ALS and non-genetic ALS. The Mendelian gene variants causing ALS are associated with a younger age of onset.^9^*De novo* mutations in *FUS* and *SOD1* have been found to be an additional cause of apparently sporadic disease.^10,11^ As genetic therapies are now being developed, and because about 15% of people with apparently sporadic ALS carry a Mendelian gene variant,^12^ genetic testing is likely to become more frequent regardless of family history.

Health resources are limited, and in some countries, testing is limited by age of onset and family history. For example, the UK National Genomic Test Directory criteria allow genetic testing only in patients with a positive family history of ALS or in those with apparently sporadic ALS with onset below 40 years of age.^13^ In other countries, testing is primarily performed by research laboratories, rather than clinical services, and there have been calls for more genetic testing availability worldwide.^14^ It remains unclear whether genetic testing should be restricted by age of onset and family history. We therefore assessed the probability of a positive genetic test result under various scenarios to provide evidence for how and when genetic testing should be performed.

## Materials and methods

### Study design and participants

People with sporadic ALS contributing to the Project MinE ALS Sequencing Consortium with relevant data available were included. Sex, age of onset and ALS phenotype were obtained for each person.

### Gene panels

We performed two analyses in the global dataset, analysing the four commonest ALS genes, usually tested clinically in an initial gene panel, and a larger panel, consisting of genes selected for harbouring likely large-effect, rare Mendelian ALS gene variants (Table 1).^1^ Further detail on the frequencies and gene burden test results are available on the ProjectMinE databrowser.^15^ The analysis was repeated, limited to the UK dataset, with a set of genes that are part of UK-based genetic testing practice.

**Table 1:** Genes tested for global project MinE and UK cohorts

Four-gene panel	Larger gene panel	Genes selected from the Genomics England ALS panel
*C9orf72*	*ANG*	*ALS2* ^a^
*FUS*	*ATXN2*	*ANG*
*SOD1*	*C21orf2*	*ANXA11*
*TARDBP*	*C9orf72*	*ATXN2*
	*CHCHD10*	*C9orf72*
	*DAO*	*CHCHD10*
	*DCTN1*	*DCTN1*
	*FUS*	*ERBB4*
	*HNRNPA1*	*FIG4*
	*MATR3*	*FUS*
	*MOBP*	*HNRNPA1*
	*NEK1*	*MATR3*
	*OPTN*	*NEFH*
	*PFN1*	*OPTN*
	*SCFD1*	*PFN1*
	*SOD1*	*SETX*
	*SQSTM1*	*SIGMAR1*
	*TAF15*	*SOD1*
	*TARDBP*	*SPG11*
	*TBK1*	*SQSTM1*
	*TUB4A*	*TARDBP*
	*UBQLN2*	*TBK1*
	*VAPB*	*TUB4A*
	*VCP*	*UBQLN2*
		*VAPB*
		*VCP*

a Genes for which only pathogenic bi-allelic variants were reported.

For the analysis restricted to UK samples, 26 ALS genes were selected from the Genomics England ‘Amyotrophic lateral sclerosis/motor neuron disease v1.48’ panel (Table 1).^16^ As this panel also contained genes thought to be linked with non-ALS conditions, 13 additional genes in the official panel, not currently widely accepted to be relevant to ALS, were excluded. Furthermore, genes with common variants of small effect were also excluded, as described below.

### Whole-genome sequencing, bioinformatics, quality control and variant prioritisation

All samples used were part of Project MinE data freeze 2. Sequencing data, quality control and the analysis pipeline have been described previously.^17,18^ In brief, the case and control samples were sequenced using PCR-free library preparation on the Illumina HiSeq 2000 and HiSeq × platforms to ∼35× coverage with 100 bp reads and ∼25× coverage with 150 bp reads, respectively. Sequencing data alignment to GRCh37 and variant calling were performed using the Illumina Isaac pipeline.^19^ Sites with a genotype quality <10 and variants with low quality scores (<20 for single nucleotide variants and <30 for indels) were removed. Samples with a transition-transversion ratio, total number of single nucleotide variants, indels and singletons outside the interval mean ± 6 SD from the full distribution of samples were removed. Variants with missingness >2% across all samples were excluded. Genetically inferred sex, based on the number of X and Y chromosomes, was compared to the sex reported in the phenotypic data. Samples with mismatched sex information and missing age of onset were removed. The resulting variants were annotated using the Ensembl variant effect predictor (VEP),^20^ choosing to restrict the results to one selected allele per variant. All other VEP options were kept to default. In order to include in this study variants that would be reported as a result of genetic testing, we retained rare variants (minor allele frequency <0.001 in both gnomAD version 2.1.1 non-Finnish European data and our 2446 Project MinE control dataset) in selected ALS-relevant gene sets, with a predicted moderate and high functional impact on gene function as defined by VEP. In brief, using consequence terms from the sequence ontology,^21^ moderate impact variants included missense, in-frame insertions and deletions and protein altering variants. High impact variants included stop lost and gained, start lost, transcript amplification, frameshift, transcript ablation and splice acceptor and donor variants.

### *C9orf72* and *ATXN2* expansion testing

We used ExpansionHunter to estimate the number of *C9orf72* GGGGCC hexanucleotide and *ATXN2* CAG trinucleotide repeats in the sequence data. ExpansionHunter has been previously validated for the detection of hexanucleotide and trinucleotide repeat expansions in a number of studies, showing a detection accuracy of >99%.^22–24^ For the *C9orf72* expansion, we considered >30 repeats to be pathogenic.^25^ For the *ATXN2* expansion, we defined as pathogenic an intermediate expansion (known to be an ALS risk factor) with repeat counts of 29–33 inclusive.^26^

### Automatic application of the ACMG guidelines

The classification of the variants into benign, likely benign, variant of uncertain significance (VUS), likely pathogenic, or pathogenic variants, according to the American College of Medical Genetics (ACMG) guidelines,^29^ was done using InterVar (version 2.2.2).^30^ The ACMG guidelines are based on 28 criteria (16 for pathogenicity and 12 for benignity), 18 of which can be evaluated by InterVar automatically. In a real clinical setting, the application of the remaining 10 criteria would require a manual review of all supporting evidence available from literature and public databases for each individual variant. However, given the large scale of this project, we approximated this process by using sets of fixed values for the moderate and high impact variants in our analyses (see Supplementary Table 1 for each criterion, values selected and their rationale). InterVar was then used to classify the variants according to ACMG guidelines by applying 18 automatically evaluated criteria with default parameters and using two sets of manually predefined values for the 10 remaining criteria. VUSs were further classified into ‘high probability of pathogenicity’ if they matched any of the 16 criteria of pathogenicity and did not match any of the 12 criteria of benignity, and ‘low to medium probability’ otherwise.

### Definition of a clinically actionable result

A clinically actionable genetic test result was defined as one reporting variants with a predicted moderate or high functional impact as predicted by VEP, not classified as benign or likely benign, and a VUS of ‘low to medium probability of pathogenicity’, or one reporting a pathogenic repeat size in the *C9orf72* or *ATXN2* genes. In the gene panels, some genes, such as *NEK1* and *OPTN*, have both heterozygous and homozygous risk alleles, and, in these cases, we retained them as reportable when a single heterozygous variant was identified. For *ALS2*, only homozygosity has been associated with ALS, and we therefore report recessive frequencies. To show that our automated definition of pathogenicity was reasonable, we repeated the four-gene analysis using widely-accepted database definitions of pathogenicity: *C9orf72* repeat expansion >30; all rare *SOD1* variants, based on the assumption that all such classes are pathogenic; and, for *TARDBP* and *FUS*, all rare variants classified as being pathogenic or likely pathogenic in the ClinVar database or present in the ALS online database (ALSoD), having been reported in at least one publication and ≥2 patients.^27,28^

### Statistical analysis

Patients were grouped by age of disease onset: <40, 40–49, 50–59, 60–69 and ≥70 years. The proportion who were carriers of a clinically actionable variant was estimated for each group, along with a 95% confidence interval (CI).

#### Calculation of incidence rates of ALS for the UK by age of onset and sex

We used population-based data from The National MND Register of England, Wales and Northern Ireland.^31^ Incidence rates of ALS were calculated for males and females grouped by age of onset. The catchment area of the population was the aggregate catchment area of 15 specialist UK Motor Neuron Disease Care and Research Centres covering an area of England with a population of 12.5 million people.^31,32^ People with a date of diagnosis of ALS between 1 Jan 2018 and 31 Dec 2019 were included. Direct standardization, which refers to the weighting of crude incidence rates by a reference population (in this case, the population structure of the UK using 2011 Office of National Statistics census data),^33^ was achieved by multiplying the crude incidence rate by the number of people in the standard population for that age and sex group. The 95% confidence intervals of the directly standardized rates were calculated using the exact method by approximating from a gamma distribution.^34^ As the population included people with both apparently sporadic and familial ALS, and given that 5–20% of people with ALS report a family history, expected incidence rates of ALS were multiplied by a factor of 0.875 (halfway between 80 and 95%) to estimate incidence rates specific for sporadic ALS. Data were analysed in R version 4.0.2 with package ‘epitools’.^35^

#### Estimation of the number and proportion of cases missed by limiting genetic testing by age of onset

Given that the UK’s current National Genomic Test Directory criteria use an age of onset of <40 years as the cut-off for ALS genetic testing in those without a family history, we estimated the number of people with sporadic ALS that would be missed in the UK each year by limiting testing to those below the age of 40 years. This was calculated by multiplying the age and sex-specific probabilities of having a clinically actionable genetic test result by the age and sex-specific incidence rates of ALS for the UK and summing these quantities across all age groups ≥40 years. The proportion of missed clinically actionable tests was calculated by multiplying the estimated number of missed sporadic cases in each age interval ≥40 years by the proportion of ALS patients with a clinically actionable test in the corresponding age interval. The 95% confidence intervals were calculated.

### Data availability

Individual whole-genome sequencing data are available and can be requested through Project MinE (https://www.projectmine.com/research/data-sharing/). A data access committee controls access to raw data, ensuring a FAIR data setup (https://www.datafairport.org).

## Results

### Global Project MinE cohort

**Figure 1:**
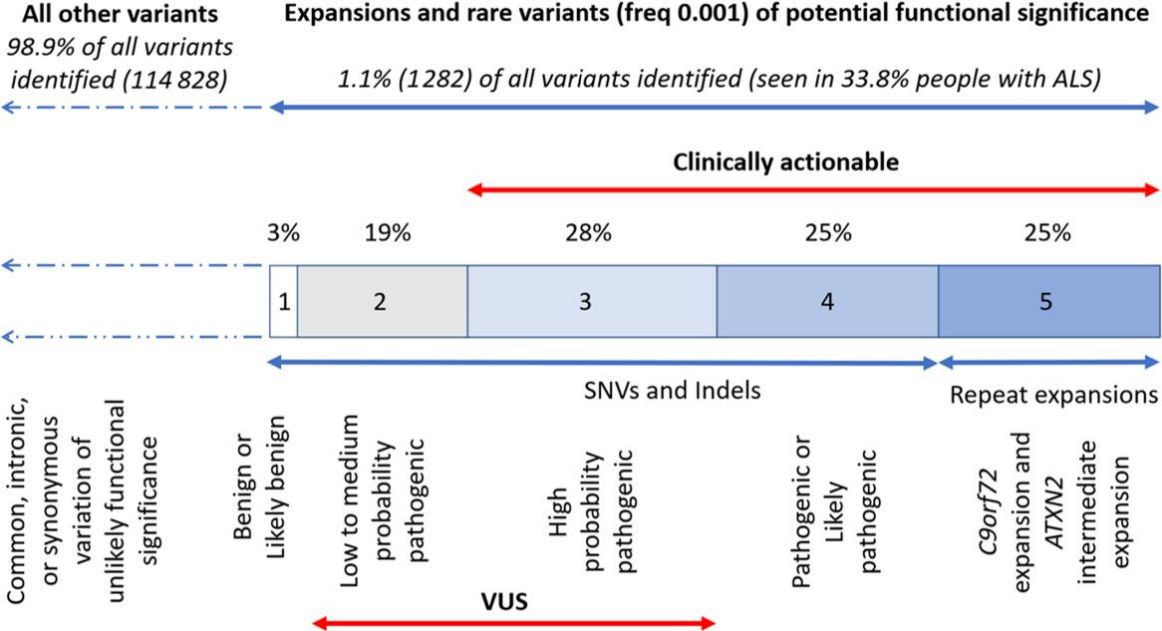
Distribution of identified variants in the Global Project MinE cohort and their classification by ACMG criteria.

There were 6274 patients with sporadic ALS. Samples were from the following countries: Belgium (*n* = 547), France (*n* = 149), Ireland (*n* = 465), Israel (*n* = 104), Italy (*n* = 61), Netherlands (*n* = 1673), Portugal (*n* = 59), Spain (*n* = 381), Sweden (*n* = 194), Switzerland (*n* = 53), Turkey (*n* = 602), UK (*n* = 1551) and USA (*n* = 435). ALS was defined using the Gold Coast criteria,^36^ with the addition of primary lateral sclerosis because of the difficulty in reliably distinguishing this variant from ALS in the first 3 years. Restricting the analysis to ALS defined by El Escorial criteria definite, probable and laboratory-supported probable did not alter the findings (Supplementary Fig. 1). Data for *UBQLN2* on chromosome X was available for 73% of ALS cases. There were 116 110 variants identified, of which 1.1% were rare variants of potential functional significance or repeat expansions in *C9orf72* or *ATXN2* (Fig. 1). Based on ACMG criteria, 47% of these were VUS. We defined 78% as clinically actionable, comprising 28% high probability pathogenic VUS, likely pathogenic or pathogenic variants, and pathological repeat expansions in *C9orf72* and *ATXN2*.

**Figure 2:**
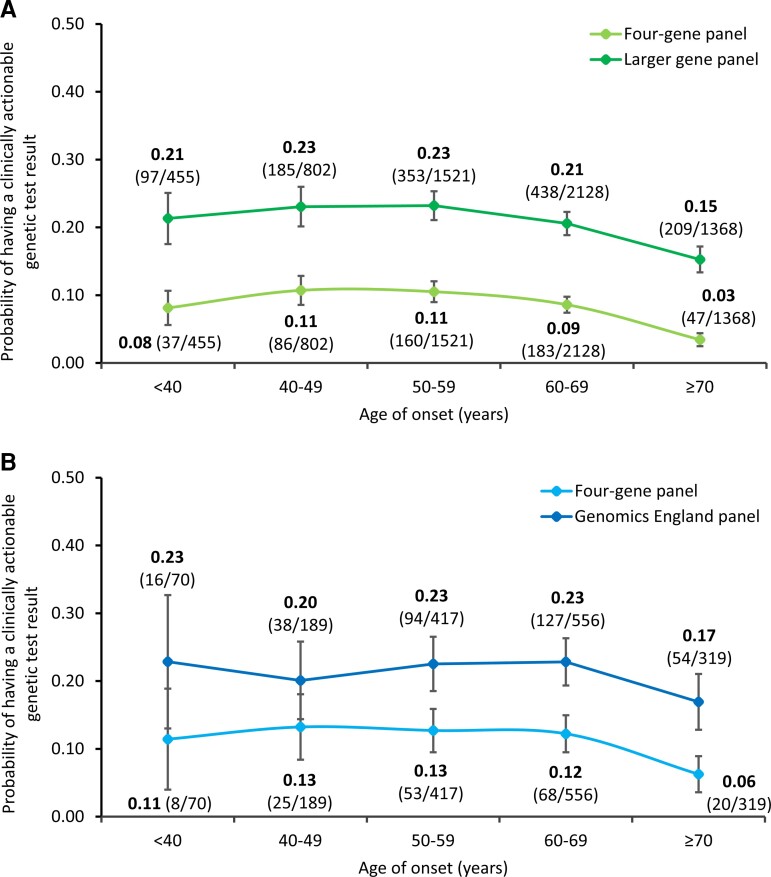
**Probability of a person with ALS having a clinically actionable genetic test result given their age of onset**. (**A**) Global Project MinE cohort. (**B**) UK cohort. Error bars denote 95% CI.

For people with apparently sporadic ALS, 8% (*n* = 513) had a clinically actionable result in the four-gene panel and 20% (*n* = 1282) in the larger gene panel. The probability of a clinically actionable result was high in the youngest age category (<40 years age of onset group), with 8% having such an outcome in the four-gene panel and 21% in the larger gene panel (Fig. 2A and Supplementary Fig. 1A) but not always maximal in this age group. Those in the oldest age category (≥70 years age of onset group) still had a substantial risk, with 3% having a clinically actionable outcome in the four-gene panel and 15% in the larger gene panel.

### UK cohort

There were 1551 people with sporadic ALS from the UK. Of these, 11% (*n* = 174) had a clinically actionable genetic test result using the four-gene panel, and 21% (*n* = 329) for the Genomics England panel. Data for *UBQLN2* was available for 89% of UK ALS cases. The probability of a clinically actionable genetic test result was high for the youngest age group (<40 years age of onset) at 11% for the four-gene panel and 23% for the Genomics England panel (Fig. 2B and Supplementary Fig. 1B) but again, not always maximal in this age group. Furthermore, there remained a substantial risk for those aged ≥70 years at 6% for the four-gene panel and 17% for the Genomics England panel.

### ClinVar and ALSoD corroboration of pathogenicity definition

Across all age groups, 8% (*n* = 513) would have a clinically actionable test using the four-gene panel on our global cohort. Using the ClinVar and ALSoD method for the same four genes, this is 7% (*n* = 454), suggesting that our automated definition of pathogenicity is reasonable.

### Number of cases with clinically actionable genetic results missed per year in the UK

Table 2 contains age and sex-specific incidence rates of ALS, calculated per 100 000 person-years, as well as the expected number of new sporadic ALS diagnoses in the UK per year that have been adjusted to the UK population structure. Restriction of testing by age in apparently sporadic ALS results in 56 (31 male, 25 female) of 58 people with clinically actionable results being missed using the four-gene panel (0.97, 95% CI 0.92–1.01) and 115 (63 male, 52 female) of 117 people being missed using the Genomics England panel (0.98, 95% CI 0.96–1.01) each year in the UK, representing 97 to 98% of clinically actionable sporadic ALS genetic test results being missed using this policy.

**Table 2:** Age- and sex-specific incidence rates per 100 000 person-years in the UK, and age- and sex-specific expected number of new sporadic ALS diagnoses in the UK per year

Age of onset, years	Incidence rates of ALS per 100 000 person-years in the UK		Expected number of new sporadic ALS diagnoses per year in the UK		
	Male	Female	Male	Female	Total
<40	0.080 (0.022–0.20)	0.020 (0.0005–0.011)	7.03 (1.92–18.01)	1.78 (0.045–9.91)	8.81 (1.96–27.92)
40–49	0.89 (0.54–1.38)	0.40 (0.18–0.75)	35.74 (21.83–55.19)	16.24 (7.43–30.83)	51.98 (29.25–86.02)
50–59	2.67 (1.98–3.53)	0.80 (0.45–1.32)	89.11 (65.92–117.81)	27.35 (15.31–45.12)	116.46 (81.23–162.92)
60–69	3.19 (2.38–4.20)	2.91 (2.15–3.85)	93.03 (69.27–122.32)	89.05 (65.88–117.73)	182.08 (135.15–240.05)
≥70	3.65 (2.75–4.73)	3.20 (2.48–4.08)	99.90 (75.46–129.73)	118.52 (91.66–150.79)	218.42 (167.13–280.51)

Values are presented as incidence rate (95% CI).

## Discussion

We have shown that there is a substantial risk of a clinically actionable genetic test result in ALS, regardless of age of onset and family history, with 15 to 17% of those with apparently sporadic disease aged 70 years or over having a clinically actionable result on widely used ALS gene panels. Using UK guidelines as an example of typical practice, we estimate that restriction of testing in those without a family history to people aged 40 years or younger means up to 115 people with a clinically actionable result per year, and 97–98% of clinically actionable sporadic ALS results will be missed in the UK. Extrapolating this to other health systems, each year, thousands of people with ALS will remain undetected as having a clinically actionable genetic test result simply as a result of guidelines rather than availability of access.

Until recently, the main clinical benefit of genetic testing in ALS was for family planning and the possibility of preimplantation genetic diagnosis. Now, however, there are several clinical trials in progress targeting people with variations in specific genes. A childhood motor neuron disease, spinal muscular atrophy, can be successfully treated with gene therapy, and related approaches in adult motor neuron diseases such as ALS have shown promising results in Phase 2 clinical trials.^4^ Identifying clinically actionable genetic test results in someone with ALS, an otherwise uniformly fatal disease, is therefore crucial as a means of opening up new therapeutic approaches.

Our findings build on previous work showing that familial ALS is not the same as genetic ALS, and that the distinction between familial and sporadic disease is based on an erroneous assumption that genetic variation does not contribute to sporadic ALS.^8^ Genotyping of sequential ALS patients regardless of family history shows that 21% carry a pathogenic variant, with 93% having no family history of ALS, and 15% meeting the inclusion criteria for a current ALS gene therapy clinical trial.^12^ We have extended this to show the need to sequence people of all ages. The presence of pathogenic variants in older apparently sporadic ALS patients is unsurprising if we consider that family history in preceding generations may be less apparent when life expectancy was lower, resulting in an ascertainment bias. Lifting of age restrictions for genetic testing is not only important for identifying those affected in older age groups, but also for pulling out at-risk relatives in future generations, allowing for them to be identified before they present clinically. This early diagnosis in future generations is likely to be important as we learn more about when we should intervene with gene therapies, as the point of first presentation may be too late. We recommend that we should be following families carrying known variants carefully, enrolling them in pre-symptomatic studies, with a view to offering preventative treatment once more is known about the optimal time point at which we should intervene.

Another consequence of using an arbitrary age limit for offering genetic testing is that our ability to build a knowledge base of the relevance of variants of uncertain significance in ALS will be severely hampered by missing data, lending more support to our view that testing should be unrestricted by age and family history.

As the number of accepted ALS genes increases and testing panels increase in size, the percentage of clinically actionable results will rise, and the number of missed results will be even greater than currently. However, expanding the availability of genetic testing will inevitably need to be carefully balanced with appropriate resources for counselling and for this to be delivered by the most appropriate professional, which may be the clinician in tertiary care specialist services.

A limitation of this study is in the definition of a clinically actionable result. Curation of disease-causing genetic variation is challenging, and, with current technology, only possible for variants with multiple lines of convergent evidence. As a result, some variants we have defined as clinically actionable may be variants of uncertain significance. Given our study included whole-genome sequencing samples on a large, international scale, we curated genetic variants deemed as having the potential to be pathogenically relevant as efficiently as possible, without going into detail for each variant. This was based on previously reported association with ALS, detection in cases, but not in several control databases, and bioinformatics prediction of whether the mutation would be likely to have a functional impact on gene function. However, using ClinVar and ALSoD databases to define pathogenicity gave similar probabilities of having a clinically actionable result when testing the four commonest genes, providing confidence that our methods and results can be generalized. Ideally, determining the clinical significance of each variant would be preferable, and ongoing efforts, such as the ClinGen Amyotrophic Lateral Sclerosis Spectrum Disorders Gene Curation Expert Panel, a National Institutes of Health (NIH)-funded resource dedicated to building a central resource that defines the clinical relevance of genes and variants for use in precision medicine and research, will be essential in the future.^37^ As new variants are found and curated, our clinical model will need to be updated. A further limitation of our study is that we may have underestimated the number of clinically actionable results, owing to occasions where patients have genetic testing arranged through regular channels, but later, after testing positive, elect not to participate in further genetic testing such as part of the Project MinE ALS Sequencing Consortium and, thus, not being included. In some countries, those with a known genetic basis for their ALS were actively excluded at the start of sample collection, because it was thought that there would be little to gain from further sequencing.

In summary, this large, global study, combining both genetics and epidemiology, provides robust evidence to recommend that genetic testing in ALS should not be restricted by age of onset or family history. Instead, with increasing gene therapies on the horizon and potential for precision medicine, the gold standard should be to offer genetic testing to all patients with apparently sporadic ALS, regardless of their age of onset.

## Supplementary Material

awac279_Supplementary_Data

## Appendix 1

### Project MinE ALS Sequencing Consortium group authors

Nazli A. Başak, Philippe Corcia, Philippe Couratier, Mamede de Carvalho, Vivian Drory, Jonathan D. Glass, Marc Gotkine, John E. Landers, Russell McLaughlin, Jesus S. Mora Pardina, Karen E. Morrison, Monica Povedano, Christopher E. Shaw, Pamela J. Shaw, Vincenzo Silani, Nicola Ticozzi, Philip Van Damme, Leonard H. van den Berg, Jan H. Veldink, Patrick Vourc’h, Markus Weber.

## References

[1] Brown RH , Al-Chalabi A. Amyotrophic lateral sclerosis. N Engl J Med. 2017;377:162–172. 28700839 10.1056/NEJMra1603471

[2] Johnston CA , Stanton BR, Turner MR, et al Amyotrophic lateral sclerosis in an urban setting: a population based study of inner city London. J Neurol. 2006;253:1642–1643. 17219036 10.1007/s00415-006-0195-y

[3] Al-Chalabi A , Calvo A, Chio A, et al Analysis of amyotrophic lateral sclerosis as a multistep process: a population-based modelling study. Lancet Neurol. 2014;13:1108–1113. 25300936 10.1016/S1474-4422(14)70219-4 PMC4197338

[4] Miller T , Cudkowicz M, Shaw PJ, et al Phase 1–2 trial of antisense oligonucleotide tofersen for *SOD1* ALS. N Engl J Med. 2020;383:109–119. 32640130 10.1056/NEJMoa2003715

[5] van Eijk RPA , Jones AR, Sproviero W, et al Meta-analysis of pharmacogenetic interactions in amyotrophic lateral sclerosis clinical trials. Neurology. 2017;89:1915–1922. 28978660 10.1212/WNL.0000000000004606 PMC5664299

[6] Ryan M , Heverin M, Doherty MA, et al Determining the incidence of familiality in ALS: a study of temporal trends in Ireland from 1994 to 2016. Neurol Genet. 2018;4:e239. 29845113 10.1212/NXG.0000000000000239 PMC5961194

[7] Al-Chalabi A , Lewis CM. Modelling the effects of penetrance and family size on rates of sporadic and familial disease. Hum Hered. 2011;71:281–288. 21846995 10.1159/000330167

[8] Al-Chalabi A . Perspective: don’t keep it in the family. Nature. 2017; 550:S112. 29045374 10.1038/550S112a

[9] Mehta PR , Jones AR, Opie-Martin S, et al Younger age of onset in familial amyotrophic lateral sclerosis is a result of pathogenic gene variants, rather than ascertainment bias. J Neurol Neurosurg Psychiatry. 2019;90:268–271. 30270202 10.1136/jnnp-2018-319089 PMC6518463

[10] Kim YE , Oh KW, Kwon MJ, et al De novo FUS mutations in 2 Korean patients with sporadic amyotrophic lateral sclerosis. Neurobiol Aging. 2015;36:1604.e17–1604.e19. 10.1016/j.neurobiolaging.2014.10.002 25457557

[11] Müller K , Oh KW, Nordin A, et al De novo mutations in *SOD1* are a cause of ALS. J Neurol Neurosurg Psychiatry. 2022;93:201–206. 34518333 10.1136/jnnp-2021-327520 PMC8784989

[12] Shepheard SR , Parker MD, Cooper-Knock J, et al Value of systematic genetic screening of patients with amyotrophic lateral sclerosis. J Neurol Neurosurg Psychiatry. 2021;92:510–518. 33589474 10.1136/jnnp-2020-325014 PMC8053339

[13] National Genomic Test Directory . Testing Criteria for Rare and Inherited Disease. Accessed 25 November 2021. https://www.england.nhs.uk/wp-content/uploads/2018/08/Rare-and-inherited-disease-eligibility-criteria-2021-22-v2.pdf

[14] Salmon K , Kiernan MC, Kim SH, et al The importance of offering early genetic testing in everyone with amyotrophic lateral sclerosis. Brain. 2022; 145:1207–1210. 35020823 10.1093/brain/awab472 PMC9129091

[15] Project MinE Data Browser . Accessed 2 June 2022. http://databrowser.projectmine.com/

[16] Genomics England . Amyotrophic lateral sclerosis/motor neuron disease (Version 1.49). Accessed 25 November 2021. https://panelapp.genomicsengland.co.uk/panels/263/

[17] van Rheenen W , van der Spek RAA, Bakker MK, et al Common and rare variant association analyses in amyotrophic lateral sclerosis identify 15 risk loci with distinct genetic architectures and neuron-specific biology. Nat Genet. 2021;53:1636–1648. 34873335 10.1038/s41588-021-00973-1 PMC8648564

[18] Project MinE ALS Sequencing Consortium . Project MinE: study design and pilot analyses of a large-scale whole-genome sequencing study in amyotrophic lateral sclerosis. Eur J Hum Genet. 2018;26:1537–1546. 29955173 10.1038/s41431-018-0177-4 PMC6138692

[19] Raczy C , Petrovski R, Saunders CT, et al Isaac: ultra-fast whole-genome secondary analysis on illumina sequencing platforms. Bioinformatics. 2013;29:2041–2043. 23736529 10.1093/bioinformatics/btt314

[20] McLaren W , Gil L, Hunt SE, et al The ensembl variant effect predictor. Genome Biol. 2016;17:122. 27268795 10.1186/s13059-016-0974-4 PMC4893825

[21] Eilbeck K , Lewis SE, Mungall CJ, et al The sequence ontology: a tool for the unification of genome annotations. Genome Biol. 2005;6:R44. 15892872 10.1186/gb-2005-6-5-r44 PMC1175956

[22] Dolzhenko E , van Vugt J, Shaw RJ, et al Detection of long repeat expansions from PCR-free whole-genome sequence data. Genome Res. 2017;27:1895–1903. 28887402 10.1101/gr.225672.117 PMC5668946

[23] Tazelaar GHP , Boeynaems S, De Decker M, et al *ATXN1* Repeat expansions confer risk for amyotrophic lateral sclerosis and contribute to TDP-43 mislocalization. Brain Commun. 2020;2:fcaa064. 32954321 10.1093/braincomms/fcaa064 PMC7425293

[24] Iacoangeli A , Al Khleifat A, Jones AR, et al C9orf72 intermediate expansions of 24–30 repeats are associated with ALS. Acta Neuropathol Commun. 2019;7:115. 31315673 10.1186/s40478-019-0724-4 PMC6637621

[25] DeJesus-Hernandez M , Mackenzie IR, Boeve BF, et al Expanded GGGGCC hexanucleotide repeat in noncoding region of C9ORF72 causes chromosome 9p-linked FTD and ALS. Neuron. 2011;72:245–256. 21944778 10.1016/j.neuron.2011.09.011 PMC3202986

[26] Sproviero W , Shatunov A, Stahl D, et al ATXN2 Trinucleotide repeat length correlates with risk of ALS. Neurobiol Aging. 2017;51:178.e1–178.e9. 10.1016/j.neurobiolaging.2016.11.010 PMC5302215 28017481

[27] Landrum MJ , Chitipiralla S, Brown GR, et al Clinvar: improvements to accessing data. Nucleic Acids Res. 2020;48:D835–D844. 31777943 10.1093/nar/gkz972 PMC6943040

[28] Abel O , Powell JF, Andersen PM, Al-Chalabi A. ALSoD: a user-friendly online bioinformatics tool for amyotrophic lateral sclerosis genetics. Hum Mutat. 2012;33:1345–1351. 22753137 10.1002/humu.22157

[29] Richards S , Aziz N, Bale S, et al Standards and guidelines for the interpretation of sequence variants: a joint consensus recommendation of the American college of medical genetics and genomics and the association for molecular pathology. Genet Med. 2015;17:405–424. 25741868 10.1038/gim.2015.30 PMC4544753

[30] Li Q , Wang K. Intervar: clinical interpretation of genetic variants by the 2015 ACMG-AMP guidelines. Am J Hum Genet. 2017;100:267–280. 28132688 10.1016/j.ajhg.2017.01.004 PMC5294755

[31] Opie-Martin S , Ossher L, Bredin A, et al Motor neuron disease register for England, Wales and Northern Ireland-an analysis of incidence in England. Amyotroph Lateral Scler Frontotemporal Degener. 2021;22:86–93. 32940088 10.1080/21678421.2020.1812661

[32] Office for National Statistics . 2011 Census: Additional detailed and local characteristics tables for England and Wales (part 2). LC1117EW—Sex by age. Accessed 25 November 2021. https://www.ons.gov.uk/peoplepopulationandcommunity/populationandmigration/populationestimates/datasets/2011censusadditionaldetailedandlocalcharacteristicstablesforenglandandwalespart2

[33] Office for National Statistic . 2011 Census: Population estimates by single year of age and sex for local authorities in the United Kingdom. Accessed 25 November 2021. https://www.ons.gov.uk/peoplepopulationandcommunity/populationandmigration/populationestimates/datasets/2011censuspopulationestimatesbysingleyearofageandsexforlocalauthoritiesintheunitedkingdom

[34] Fay MP , Feuer EJ. Confidence intervals for directly standardized rates: a method based on the gamma distribution. Stat Med. 1997;16:791–801. 9131766 10.1002/(sici)1097-0258(19970415)16:7<791::aid-sim500>3.0.co;2-#

[35] Aragon TJ . epitools: Epidemiology Tools. 2020.

[36] Shefner JM , Al-Chalabi A, Baker MR, et al A proposal for new diagnostic criteria for ALS. Clin Neurophysiol. 2020;131:1975–1978. 32387049 10.1016/j.clinph.2020.04.005

[37] Clinical Genome Resource . Amyotrophic Lateral Sclerosis Spectrum Disorders Gene Curation Expert Panel. Accessed 25 November 2021. https://clinicalgenome.org/affiliation/40096/

