# Supplementary material for: The impact of age on genetic testing decisions in amyotrophic lateral sclerosis
Source: Brain. 2022 Sep 27;145(12):4440–7. doi: 10.1093/brain/awac279 (PMC9762932; doi:10.1093/brain/awac279)
Supplement: awac279_Supplementary_Data [file awac279_supplementary_data.pdf]

Supplementary Material

Supplementary Figure 1: Probability of a person with ALS (restricting the analysis to ALS defined by El Escorial criteria definite, probable, and laboratory probable) having a clinically actionable genetic test result given their age of onset. (A) Global Project MinE cohort. **(B)** UK cohort. Error bars denote 95% confidence intervals.

Supplementary Figure 1

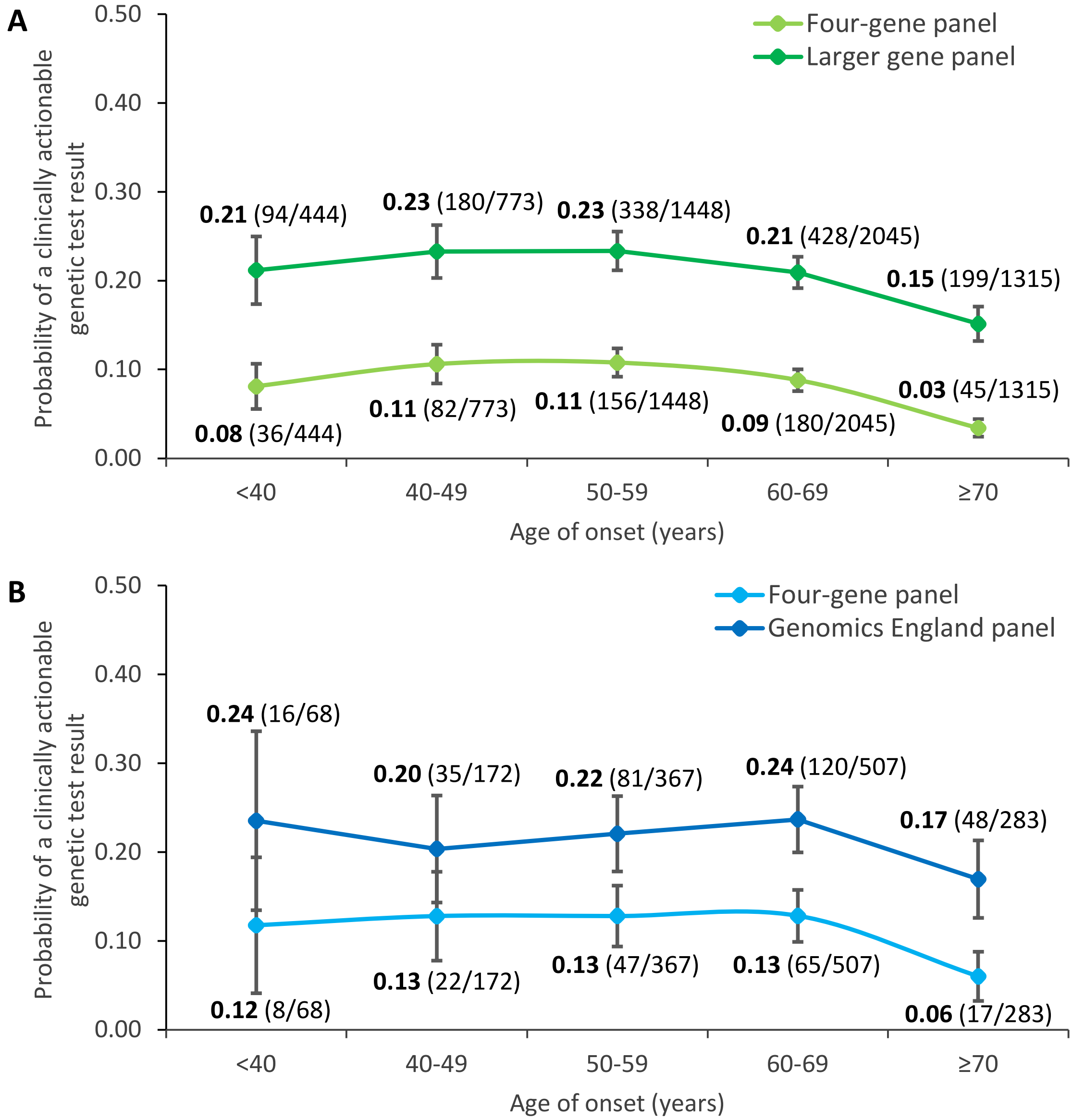

Supplementary Table 1: Manual setting of ACMG criteria for the InterVar annotation.

Criterion ID	Criterion Description	Values (high impact)	Values (moderate impact)	Rationale
PS2	Confirmed *de novo* in patient with the disease and no family history	0	0	Although the patients in this study were sporadic, we could not confirm if the variants were *de novo* as parents' data was not available and complete penetrance cannot be assumed
PM6	Assumed *de novo*	0	0	Same as above
PS3	Established *in vitro* or *in vivo* functional studies supportive of a damaging effect on the gene or gene product	1	0	It is reasonable to assume that high impact variants (mostly protein truncating variants) affect the gene or gene product while moderate effect variants (mostly missense) do not
BS3	Established *in vitro* or *in vivo* functional studies show no damaging effect on protein function or splicing	0	0	We could not confirm this without reviewing each variant individually. However, such studies would not exist for the great majority of variants in this study
PM3	For recessive disorders, detected in trans with a pathogenic variant	0	0	Variants are assumed dominant. Exclusively recessive genes were excluded and the great majority of other variants show dominant patterns of inheritance
BP2	Observed in trans with a pathogenic variant for a fully penetrant dominant gene/disorder; or observed in cis with a pathogenic variant in any inheritance pattern	0	0	Variants were assessed individually. Not relevant for our study as the focus is the proportion of patients with a clinically actionable variant
PP1	Co-segregation with disease in multiple affected family members in a gene definitively known to cause the disease	0	0	Not applicable as necessary data was not available
PP4	Patient's phenotype or family history is highly specific for a disease with a single genetic aetiology	1	1	Genes tested are all specific for ALS
BP5	Variant found in a case with an alternate molecular basis for disease	0	0	Variants were assessed individually. Not relevant for our study as the focus is the proportion of patients with a clinically actionable variant
